# Towards a safe and effective chlamydial vaccine: Lessons from the eye

**DOI:** 10.1016/j.vaccine.2013.10.016

**Published:** 2014-03-20

**Authors:** David C.W. Mabey, Victor Hu, Robin L. Bailey, Matthew J. Burton, Martin J. Holland

**Affiliations:** Department of Clinical Research, Faculty of Infectious and Tropical Diseases, London School of Hygiene & Tropical Medicine, United Kingdom

**Keywords:** Chlamydia, Vaccine, Trachoma, Protective immunity, Immunopathology

## Abstract

As well as being the most common bacterial sexually transmitted infection, *Chlamydia trachomatis* (*Ct*) is the leading infectious cause of blindness. The pathogenesis of ocular chlamydial infection (trachoma) is similar to that of genital infection. In the 1960s the efficacy of *Ct* vaccines against ocular infection was evaluated in major field trials in Saudi Arabia, Taiwan, The Gambia, India and Ethiopia. These trials showed that it was possible to induce short term immunity to ocular infection, and to reduce the incidence of inflammatory trachoma, by parenteral immunisation with killed or live whole organism vaccines. In one study, it was also shown that the incidence of scarring sequelae was reduced in vaccinated children. Detailed studies in non-human primates conducted at this time suggested that vaccination could lead to more severe inflammatory disease on subsequent challenge. Since that time there have been many studies on the immunological correlates of protective immunity and immunopathology in ocular *Ct* infection in humans and non-human primates, and on host genetic polymorphisms associated with protection from adverse sequelae. These have provided important information to guide the development and evaluation of a human *Ct* vaccine.

## Highlights

• *Chlamydia trachomatis* is the leading infectious cause of blindness.
• Vaccine trials against ocular *C. trachomatis* infection were conducted in the 1960s.
• Whole organism vaccines induced short-lived, strain-specific protective immunity.
• Many correlates of protective immunity and pathology have been defined.
• Important lessons have been learned to inform the development of a chlamydial vaccine.

## 1 Introduction

*Chlamydia trachomatis* (*Ct*) is the commonest bacterial sexually transmitted infection [1]. Because a high proportion of infected people have no symptoms, screening programmes for those at risk have been the mainstay of control programmes in countries where it is prioritised and economically sustainable. However, these programmes have failed to reduce the number of reported cases, and it has even been suggested that early detection and treatment of chlamydial infection increases its incidence by preventing the development of protective immunity [2]. A vaccine against *Ct* would be of great public health benefit.

Several reviews have summarised the evidence for protective immunity to chlamydial infection, and the immunological correlates of protective immunity and immunopathology, in a variety of animal models [3–9]; but the relevance of these to human disease is not clear. The evidence for protective immunity, natural history and immunobiology of genital *Ct* infection in humans have also been extensively reviewed [10,11]. The authors concluded that more prospective studies in women with genital chlamydial infection are needed to inform development of a safe and effective chlamydial vaccine, but pointed out that these are logistically and ethically very difficult to do [5,11].

*C. trachomatis* also infects the human eye, causing trachoma, the leading infectious cause of blindness [12–14]. The genomes of *Ct* strains isolated from the eye and genital tract are more than 99% identical [15], and the clinical and pathological findings of ocular and genital infection are similar. Infections are often asymptomatic at both sites, and are characterised by inflammation and the presence of sub-epithelial lymphoid follicles. The damage in both the eye and genital tract results from fibrosis, which progresses slowly (over months or years) at the site of inflammation.

The eye is more accessible to examination and sampling than the urethra, cervix or fallopian tubes. There is an extensive literature on the natural history, immunology and pathogenesis of human ocular *Ct* infection. Human challenge studies, detailed studies on the natural history, pathogenesis and immune response to experimental ocular infection in humans and non-human primates, and the results of several major trachoma vaccine trials in humans were reported in the 1960s. More recently there have been many publications on the immunological correlates of protective immunity and immunopathology following ocular *Ct* infection in humans, on the genetics of susceptibility to the scarring sequelae of ocular infection, and on gene expression at the site of infection in the conjunctival epithelium [16]. The purpose of this review is to summarise the state of knowledge concerning the natural history, immunology and pathogenesis of ocular *Ct* infection in humans and non-human primates (NHPs), for the benefit of those interested in the development of a vaccine against *Ct*; and to suggest how a chlamydial vaccine might be evaluated in humans.

## 2 Natural history of human ocular *C. trachomatis* infection

Human volunteer studies showed that the follicular keratoconjunctivitis characteristic of trachoma develops within 2–15 days of inoculation, depending on the dose inoculated, and resolves over several months [17,18]. The follicles of trachoma are best seen in the conjunctiva of the everted upper eyelid (the subtarsal conjunctiva) and, according to the World Health Organisation case definition, follicular trachoma (TF) is present when more than 5 follicles of >0.5 mm diameter are seen in the central area of the subtarsal conjunctiva. In some cases the follicles are accompanied by intense inflammation which obscures the conjunctival blood vessels; intense trachoma (TI) is diagnosed when more than half the blood vessels are obscured by inflammation. *Ct* bacterial loads are highest in those with TI [19]. The presence of TF and/or TI defines active trachoma. *Ct* can often be isolated from cases of active trachoma but, because follicles can persist for months or years after the infection has resolved, even the most sensitive nucleic acid detection systems often fail to identify infection in subjects with active trachoma.

Some, but not all cases of active trachoma develop conjunctival scarring, but this process usually takes several years. *Ct* cannot usually be isolated from subjects with scarring trachoma. In human volunteer studies, and in experimental infections in non-human primates, scarring sequelae were not seen following a single infection [20–24]. In trachoma endemic communities, the prevalence of scarring increases with age. It is more common in women, who are more frequently in contact with young children (the main reservoir of infection). People with intense inflammatory trachoma and persistent or recurrent *Ct* infection are more likely to develop scarring [25,26]. As the scarring progresses and the scars contract, the lashes may turn inward and rub against the cornea (trachomatous trichiasis, or TT), which is painful and causes corneal damage that may result in blindness.

## 3 Protective immunity to *C. trachomatis* in humans

Experimental studies in humans and NHPs showed that re-challenge with the same strain of *Ct* results in an attenuated clinical response compared to primary infection, with a lower bacterial load [17,20,21]. In trachoma endemic communities the prevalence of ocular *Ct* infection decreases with age, and the highest bacterial loads are found in young children, suggesting that a degree of protective immunity develops following natural infection. A study in a trachoma endemic community in The Gambia, in which members of affected households were examined and tested for ocular *Ct* infection every two weeks over a 6-month period in the absence of treatment, showed that the duration of episodes of disease and of infection was age dependent. The duration of untreated infection was estimated to be approximately 15 weeks in children aged 0–4 years, and 8 weeks in older children and adults [27,28]. The estimated incidence of infection was also lower in older individuals. The conclusion from this study is that protective immunity develops following natural infection, and is associated with both a reduced incidence and a reduced duration of infection.

## 4 Trachoma vaccine trials

Experiments in baboons and in the Taiwanese monkey (*Macaca cyclops*) in the 1960s evaluated the protective efficacy of whole organism chlamydial vaccines, delivered parenterally, against ocular infection [21,29]. In both species it was shown that vaccines can provide short term, strain-specific protection against ocular *Ct* infection, which is of relatively short duration (less than 2 years). In the Taiwan monkey exposure to a different serotype led to more severe disease in vaccinated than unvaccinated animals, suggesting that vaccination could lead to immunopathological damage on subsequent exposure [21].

Large placebo-controlled human trachoma vaccine trials, using whole organisms administered by intramuscular injection, were completed in Saudi Arabia, Taiwan, The Gambia, India and Ethiopia in the 1960s [30–36]. In Saudi Arabia, two doses of a bivalent killed whole organism vaccine, or placebo, were given to children aged less than 3 years, some of whom already had trachoma. Three vaccine groups were included, who received high or low dose aqueous vaccine, or low dose vaccine with adjuvant. Less active trachoma was seen at 6 and 12 months in children receiving the low dose aqueous vaccine compared to placebo, but a higher incidence was found in those who received a higher dose. There was no difference in active trachoma or ocular *Ct* infection between vaccine and placebo arms when the results were pooled, though a reduced bacterial load (determined by counting chlamydial inclusions in conjunctival scrapings) was found in children receiving high dose aqueous vaccine and vaccine with adjuvant [30,31].

In the first trial in Taiwan four doses of a formalin-inactivated, alum-absorbed elementary body vaccine made from a local serovar C isolate, or placebo, was given to pre-school siblings of children with active trachoma over a two year period. There was less active trachoma in vaccinated children (8% vs 18%), but the protective effect was no longer seen one year after the final dose. Two subsequent trials used killed whole organism vaccine in mineral oil, given to primary school children. A bivalent vaccine, containing a Taiwanese serovar B isolate in addition to the serovar C isolate used previously serovars, reduced the incidence of active trachoma from 8.8% to 5.1%, but this difference was not significant. In a second trial, of a monovalent vaccine containing only serovar C, there was a significantly higher incidence of active trachoma in the vaccinated group, but no difference between the groups in disease severity [32,33].

In The Gambia, live vaccines were used [34]. In the first trial, the therapeutic effect of vaccination with a Gambian isolate was assessed by randomising children with clinical signs of active trachoma to receive vaccine or placebo [35]. Eight and 17 weeks after vaccination there was a significant clinical improvement in the vaccinated but not the placebo group, and the prevalence of *Ct* infection (determined by isolation in eggs) was also reduced in the vaccinated group. The protective effect was no longer seen at one year. In the second and third Gambian trials the prophylactic effect of vaccination was determined [37]. In the second trial two doses of a monovalent vaccine, made from a local isolate with a mineral oil adjuvant, were given 6 months apart. Six months after the second dose 17/118 children in the placebo group had acquired trachoma, compared to 7/117 in the vaccinated groups (*p* = 0.053). At 12 months there was no difference between the groups [37]. In the third trial two doses of a bivalent vaccine, containing two “fast killing” isolates, was given 3 weeks apart. One of these was an ocular isolate from Saudi Arabia, and the other from the USA. At 12 and 24 months there was no significant difference in the proportion of children who had acquired active trachoma between the vaccinated and placebo arms. However, at 24 months the proportion of children in the placebo group with conjunctival scarring was higher than in the vaccinated group (18/47 vs 9/55, *p* = 0.034) [37].

In the Indian trial two doses of a bivalent, formalin inactivated vaccine or placebo were given to children aged less than 5 years without signs of clinical trachoma [36]. Twelve months after the second dose 26/182 vaccinated children had developed clinical trachoma (14%), compared to 32/87 in the placebo group (37%) (*p* < 0.01). Among those who acquired trachoma, there was no difference in severity between vaccinated and control children.

These trials showed that whole organism vaccines can reduce ocular *Ct* infection and active trachoma, but that protection is short lived and, in some cases, strain-specific. Most encouragingly in The Gambia, where the presence of conjunctival scarring was also recorded, there was evidence that vaccination reduced the incidence of scarring disease. Trials in non-human primates, in particular those in the Taiwan monkey, suggested that vaccination could lead to more severe disease on subsequent exposure; but there was no convincing evidence that vaccination led to more severe disease in humans.

Since the 1960s considerable efforts have been made to develop a subunit vaccine against *Ct*, but only one of these has shown evidence of protection in a NHP [38]. *Ct* major outer membrane protein (MOMP), when given parenterally in its native form (i.e. maintaining its tertiary structure), reduced the bacterial load in cynomolgus monkeys at the time of peak shedding following ocular infection (days 3–14). However, it had no impact on the duration of infection or on the progression of clinical disease. On the other hand, a live attenuated vaccine, consisting of a plasmid-cured (P-) clinical serovar A trachoma isolate (A2497) caused a productive infection, but minimal pathology when inoculated into the eyes of cynomolgus macaques. A2497P-provided a degree of protection from infection and clinical disease on subsequent challenge with the wild type strain [39]. Three of 6 vaccinated monkeys were resistant to challenge ocular infection and, in the 3 which became infected, the bacterial load was lower than in control animals. The 3 monkeys that were protected from infection shared a common MHC class II haplotype. There was no evidence that vaccination led to more severe disease in animals which succumbed to challenge infection [39].

## 5 Immunological correlates of protective immunity and immunopathology in humans

The relationship between the immune response and clinical outcomes in human ocular *Ct* infection has been studied in longitudinal cohorts, in which subjects who clear the infection are compared with those who do not; in case-control studies comparing subjects with current infection with those with clinical signs of active trachoma in the absence of detectable *Ct* infection (who are presumed to have recently cleared their infection); and in case-control studies comparing subjects with scarring trachoma with age- and sex-matched controls from the same community, who are presumed to have had a similar lifetime exposure to ocular *Ct* infection. Among the many advantages of studying ocular infection are the unambiguous phenotype, which can be easily determined by everting the upper eyelid, and the ability to study immune responses at the site of infection in the conjunctival epithelium.

### 5.1 Cohort studies

Tear fluid or sera from children with trachoma can neutralise homologous ocular isolates of *Ct* if incubated with them before inoculation into the owl monkey eye [40]. Serovar-specific neutralising epitopes have been mapped to the MOMP [41]. However, cohort studies in trachoma endemic communities found no evidence that local anti-chlamydial IgG antibodies in ocular secretions were associated with a reduced incidence of infection. Indeed, the presence of anti-chlamydial IgG in ocular secretions was associated with an increased incidence of active trachoma in this study. The incidence was lower in subjects with anti-chlamydia IgA antibodies in ocular secretions, but the difference was not statistically significant [42]. In NHPs reduction in shedding and clearance of *Ct* infection was associated with antibody responses to a limited number of native proteins (MOMP, PmpD, Hsp60, CPAF, pgp3 and 3 as yet unidentified polypeptides) which were slowly acquired as the B cell immune response matured [43]. Children who spontaneously resolved ocular *Ct* infection had higher peripheral blood mononuclear cell (PBMC) proliferative responses to chlamydial antigens than children with persistent infection and disease [44], whereas increased conjunctival expression of *IL-10* and *FOXP3* were associated with longer episodes of infection [45].

### 5.2 Case-control studies of active trachoma

Conjunctival gene expression profiling showed that T-helper 1 (Th1) (*IFNγ*, *IL12*) cytokine expression was increased in children with active trachoma and *Ct* infection [46,47]. One study showed that the expression of *FOXP3*, a marker for T-regulatory cells, was increased in children with clinical signs of trachoma in whom infection had resolved [48]. The expression of *IL17A* is significantly increased in active trachoma [49,50]. IL17A is the signature cytokine of Th17 cells, a CD4+ T-cell population which act in an antigen-specific manner [51], but is also produced by other cell types such as γδ T-cells, NK cells, macrophages, neutrophils [52,53]. IL17A is pro-inflammatory and plays an important role in host immunity to extracellular and some intracellular pathogens. It may contribute to fibrosis through several mechanisms, including epithelial-mesenchymal transition (EMT) and increased collagen production in a TGFβ1-dependant manner [54].

In both active and scarring trachoma, conjunctival transcriptome studies showed evidence of prominent innate immune responses [49,55]. In active disease there was marked enrichment of neutrophil and NK cell related transcripts [49]. Given that NK cells are a significant source of the anti-fibrotic and anti-chlamydial cytokine IFNγ [56], have a direct anti-fibrotic role in other diseases such as cirrhosis [57], are important in maintaining the epithelial cell barrier via IL-22 production and are lytic for infected cells [58], the activity of NK cells and their interaction with adaptive T cells may be crucial in the balance between immunity and pathology [59]. Many other pathways were also differentially expressed, including pattern recognition receptors and chemokines such as neutrophil chemotactic factor *CXCL5* [50].

### 5.3 Case-control studies of scarring trachoma

Serological responses associated with scarring or protection from scarring have been identified by genome wide profiling, using an in vitro system expressing 908 open reading frames (ORFs) of the *Ct* serovar D genome and plasmid (pORF1-8)) [60]. Responses to 4 antigens were associated with trichiasis (CT414, 667, 695, 706), and to 8 antigens (CT019, 117, 301, 553, 556, 571, 709) with protection from trichiasis. These are important findings that could guide the selection of antigens to be included in a vaccine, but the results should be treated with caution, since several immunodominant antigens were not consistently recognised by the majority of sera, probably due to conformation of the antigens in the *in vitro* expression system. Moreover, antigens recognised by T- as well as B-cells are likely to be important components of a chlamydial vaccine.

Antibody responses to CT795 were associated with inflammatory trachoma, antibodies to CPAF with trichiasis [61], and antibodies to cHsp60 with scarring [62]; but it is unclear whether these antibodies have a pathogenic role or are simply markers of previous infection. Other studies have suggested that immune responses to cHsp60 may be protective: PBMC proliferation responses to cHsp60 were weaker in subjects with conjunctival scarring than in controls, while the resolution of infection was associated with increased responses [44,63].

T-helper 2 (Th2) dominated responses have been linked to fibrotic complications in some infectious diseases, e.g. schistosomiasis [64,65]. Adults with conjunctival scarring, compared to controls, have reduced lymphoproliferative responses and IFNγ production following stimulation with *Ct* EB and some chlamydial antigens, but an increased number of IL-4 producing cells in response to cHsp60 [63,66]. This provides some support for the hypothesis that individuals with scarring may have weaker Th1 cell-mediated responses to *Ct*, leading to prolonged infection and inflammation, possibly as a result of Th2 responses with pro-fibrotic effects. Studies comparing the conjunctival transcriptome by microarray and RT-PCR in subjects with scarring trachoma and matched controls found no evidence of polarisation towards Th2 responses [49,55,67,68]. Th2 cytokine levels in tear fluid were not increased in scarred individuals [69], and cytokine production in response to chlamydial antigens was no different in PBMC from cases and controls [56].

We identified a higher frequency of IL-10 [66] expression in PBMCs from cases of scarring than controls, but no differences in T regulatory cell subsets [56]. IL-10 is produced by several T cell subsets, and is not well accommodated by the T helper cell dichotomy. A case control study identified a single nucleotide polymorphisms (SNP) in the IL-10 gene that was associated with scarring [66,70–73]. Gene expression studies in the conjunctival epithelium of subjects with active trachoma who were heterozygous for a SNP in the transcribed portion of the IL-10 gene found that the haplotype associated with scarring was transcribed more efficiently than the other allele, suggesting that increased expression of IL-10 predisposes to adverse sequelae of *Ct* infection [74].

Expression of pro- inflammatory mediators such as psoriasin-1 (*S100A7*), *IL1B* and *CXCL5* is upregulated in scarring trachoma [55,68]. These factors induce neutrophil chemotaxis, and their expression was particularly increased in inflamed cases. Expression of the antimicrobial peptide *S100A7* was associated with recurrent trichiasis [75]. The importance of the chemokine response in trachoma is further supported by the finding that genetic variation across the *IL8* locus, defined by haplotypes of multiple SNPs, was associated with scarring [76]. TNF is a key cytokine in acute inflammation and has been associated with scarring trachoma in several studies: elevated levels have been found in tear fluid, and increased secretion from PBMC from scarred subjects stimulated with chlamydial elementary bodies [69,70,77,78]. Increased conjunctival transcript levels of *TNFA*, as well as *IL1B*, have also been associated with active disease and *Ct* infection [46,47,79].

Scarring develops when normal tissue architecture is disrupted and replaced by excessive connective tissue through the abnormal accumulation of extracellular matrix (ECM). Tissue damage [80] can be mediated through a variety of cell types and mechanisms. Neutrophil infiltration appears important in trachoma: neutrophils have been identified in conjunctival biopsies; produce toxic reactive oxygen and nitrogen species which damage host tissue in animal models of genital tract infection; and can produce matrix metalloproteinases (MMPs) [81,82]. The archetypal and abundant Th1 cytokine IFNγ (also produced by NK cells), considered to be central to chlamydial control, is also an inducer of MMPs [83].

The MMPs are a group of more than 25 endopeptidases with multiple, complex functions. While MMPs are required for normal tissue homeostasis, there is also evidence that they play a role in the pathogenesis of a range of inflammatory-fibrotic diseases [84–86], disrupting the basement membrane and aiding the recruitment of inflammatory cells [87]. MMPs have wide-ranging effects on inflammatory and immune processes, such as modulating chemokine activity and activation of TGFβ, IL-1β and TNF [88]. They are known to be important in a number of ocular surface diseases, and inhibition of MMP activity has been shown to reduce conjunctival scarring after glaucoma surgery [89].

MMP9 is part of the neutrophil lysosome, and mediates epithelial dissolution through degradation of type IV collagen [82]. Children with active trachoma have increased amounts of conjunctival MMP9 (determined by immunohistochemistry, zymography and gene expression analysis) [46,90]. Scarring trachoma is associated with increased expression of *MMP9* and a coding SNP that is adjacent to the active binding site of the MMP9 enzyme [46,68,91], and with differential expression of *MMPs* 7, 9, 10 and 12 and tissue inhibitor of MMP (TIMP)-1; recurrence of trichiasis after surgery is associated with an altered *MMP1/TIMP1* transcript ratio [55,67,68,92].

Scar tissue in trachoma probably originates from activated fibroblasts which are stimulated to produce collagen by profibrogenic mediators (TGF-β, PDGF, CTGF and bFGF) [50,93,94]. Chemokines have also been shown to act as fibrogenic mediators, in particular, the CC- and CXC-chemokine families, and various members of these families have been associated with scarring, including the pro-fibrogenic CCL18 [50,55,69,87].

### 5.4 Implications for a vaccine against genital *Ct* infection

Since the pathology of *Ct* infection is similar in the eye and genital tract [4,16], and both are part of the common mucosal immune system, it is likely that similar processes lead to resolution of infection and/or the development of scarring sequelae at each site. The few studies that have been conducted on the immunological correlates of protective immunity and immunopathology in human genital *Ct* infection have reached broadly similar conclusions to those of studies in the eye [10,95–97]. Local, endocervical IgA antibodies appear to be protective [95], and stronger Th-1 type cell-mediated immune responses to *Ct* antigens are seen in the peripheral blood of subjects who do not have sequelae [96,97].

An important difference between ocular and genital infection is that in the eye, the damaging sequelae occur at the site of the initial infection, the conjunctival epithelium. By contrast, in the female genital tract the major sequelae develop in the fallopian tubes and not at the cervix, which is the site of inoculation. Impairment of immunological barriers to ascending infection may explain the association between HIV infection and chlamydial PID [98]; no association has been reported between HIV and trachoma. A vaccine which prevented *Ct* infection from spreading to the upper genital tract could be effective in preventing sequelae in the female genital tract.

## 6 How would a chlamydial vaccine be evaluated in humans?

### 6.1 Vaccine efficacy

The purpose of a chlamydial vaccine is to prevent the sequelae of *Ct* infection: PID, infertility, ectopic pregnancy and blinding trachoma. An effective chlamydial vaccine could prevent primary infection, prevent re-infection, modify disease progression following infection, or reduce transmission by reducing bacterial load or the duration of infection.

Phase II studies could evaluate vaccine immunogenicity, safety and efficacy in preventing *Ct* infection in human volunteers. Human challenge experiments with *Ct* have not been reported since the ocular challenge studies more than 50 years ago, but urethral challenge studies in male volunteers may be possible; there is an extensive literature on urethral challenge of human volunteers with *Neisseria gonorrhoeae*.

The primary endpoint for phase III trials would probably be *Ct* infection. The frequency of sampling would need to be determined and, in the case of genital infection, treatment would need to be given as soon as infection was detected. In the case of ocular infection in trachoma endemic communities this would not necessarily be the case, since the recommended control strategy is annual mass treatment of endemic communities or households.

Phase IV trials could aim to evaluate vaccine efficacy in preventing PID, but this would be particularly challenging, given the difficulty in making an accurate diagnosis. Improved diagnostic tests (biomarkers or imaging) will be needed. Evaluating efficacy in preventing infertility and ectopic pregnancy would require prolonged follow up and a large sample size. Phase IV trials will be confounded by the necessity to treat subjects and their partners as soon as infection is diagnosed.

Vaccine efficacy in preventing infection, or reducing inflammation, the duration of infection or the incidence and progression of scarring could be easily evaluated in a trachoma endemic community, by frequent examination of the subtarsal conjunctiva. The incidence and progression of conjunctival scarring can be determined using an ocular microscope (slit lamp). Our recent studies have shown that confocal microscopy can identify conjunctival scarring at an early stage, before it is clinically apparent [99].

### 6.2 Vaccine safety

The evidence from trachoma vaccine trials in monkeys and humans has been interpreted as showing that vaccination can lead to more severe inflammatory disease following re-challenge with a different serovar of *Ct* As discussed above, the evidence for this from human trials is not convincing; and in the only vaccine trial in which scarring was included as an endpoint, its prevalence was reduced in the vaccinated group. Nevertheless, the spectre of an immunopathological response to chlamydial vaccination will not be easily laid to rest.

It would clearly not be possible to compare disease severity in vaccinated human volunteers and those receiving placebo, since there would be an ethical imperative to treat as soon as infection was detected. Ensuring that vaccination does not lead to more severe PID on subsequent exposure to infection will be difficult until we have better diagnostic tests. Ensuring that it does not lead to an increased incidence of infertility or ectopic pregnancy will require a large sample size and prolonged follow up. On the other hand, it would be relatively easy to study the impact of vaccination on the severity of inflammatory disease in the eye, and on the incidence or progression of scarring, through frequent examination of study subjects in trachoma endemic communities.

## 7 Conclusions

The development of a vaccine against *Ct* has been held back by the widely held belief that whole organism trachoma vaccines enhanced disease severity on subsequent ocular challenge. There is no convincing evidence of this from human vaccine trials. The evidence comes from studies in non-human primates, in whom increased inflammation was seen in vaccinated animals; but the development of scarring sequelae was not evaluated in these studies. Recent studies in trachoma endemic populations have identified new vaccine candidate antigens, immunological pathways associated with disease resolution and with progressive fibrosis, and biomarkers which predict the outcome of infection. Our understanding of pathogenesis is likely to advance rapidly now that it is possible to genetically manipulate Chlamydia [100]. This new knowledge is likely to hasten the development of a safe and effective chlamydial vaccine, which could be easily evaluated in trachoma endemic communities. Careful thought would need to be given to the recruitment of study subjects since, in communities with a high prevalence, primary infection is likely to occur in early childhood.

## Disclaimer

The authors alone are responsible for the views expressed in this article and do not necessarily represent the views, decisions or policies of the institutions with which they are affiliated.

## Conflict of interest statement

The authors declare that they have no conflict of interest.
